# Sialic acid removal from dendritic cells improves antigen cross-presentation and boosts anti-tumor immune responses

**DOI:** 10.18632/oncotarget.9419

**Published:** 2016-05-17

**Authors:** Mariana Silva, Zélia Silva, Graça Marques, Tiago Ferro, Márcia Gonçalves, Mauro Monteiro, Sandra J. van Vliet, Elodie Mohr, Andreia C. Lino, Alexandra R. Fernandes, Flávia A. Lima, Yvette van Kooyk, Teresa Matos, Carlos E. Tadokoro, Paula A. Videira

**Affiliations:** ^1^ CEDOC, NOVA Medical School/Faculdade de Ciências Médicas, Universidade Nova de Lisboa, Lisboa, Portugal; ^2^ Department of Molecular Cell Biology and Immunology, VU University Medical Center, Amsterdam, The Netherlands; ^3^ Universidade Vila Velha, Espírito Santo, Brasil; ^4^ IGC, Instituto Gulbenkian de Ciência, Oeiras, Portugal; ^5^ StemLab, Cantanhede, Portugal; ^6^ UCIBIO, Departamento Ciências da Vida, Faculdade de Ciências e Tecnologia, Universidade NOVA de Lisboa, Portugal; ^7^ CQE, Centro Química Estrutural, Instituto Superior Técnico, ULisboa, Lisboa, Portugal

**Keywords:** dendritic cells, sialic acid, antigen cross-presentation, Th1-polarization, anti-tumor immunity

## Abstract

Dendritic cells (DCs) hold promise for anti-cancer immunotherapy. However, clinically, their efficiency is limited and novel strategies to improve DC-mediated anti-tumor responses are needed. Human DCs display high content of sialic acids, which inhibits their maturation and co-stimulation capacity. Here, we aimed to understand whether exogenous desialylation of DCs improves their anti-tumor immunity. Compared to fully sialylated DCs, desialylated human DCs loaded with tumor-antigens showed enhanced ability to induce autologous T cells to proliferate, to secrete Th1 cytokines, and to specifically induce tumor cell apoptosis. Desialylated DCs showed an increased expression of MHC-I and -II, co-stimulatory molecules and an augmented secretion of IL-12. Desialylated HLA-A*02:01 DCs pulsed with gp100 peptides displayed enhanced peptide presentation through MHC-I, resulting in higher activation ofgp100_280–288_ specific CD8^+^ cytotoxic T cells. Desialylated murine DCs also exhibited increased MHC and co-stimulatory molecules and higher antigen cross-presentation via MHC-I. These DCs showed higher ability to activate antigen-specific CD4^+^ and CD8^+^ T cells, and to specifically induce tumor cell apoptosis. Collectively, our data demonstrates that desialylation improves DCs' ability to elicit T cell-mediated anti-tumor activity, due to increased MHC-I expression and higher antigen presentation via MHC-I. Sialidase treatment of DCs may represent a technology to improve the efficacy of antigen loaded-DC-based vaccines for anti-cancer immunotherapy.

## INTRODUCTION

Several studies have shown the potential of dendritic cells (DCs) as therapeutic vaccination against cancer.[1–4] However, only few vaccines have been approved for clinical use.[5] One of the challenges has been the establishment of efficient approaches that enhance the maturation profile of DCs and, in particular, presentation of antigens to cytotoxic T cells.[6] In fact, efficient induction of anti-tumor responses requires that DCs undergo proper maturation. This process is characterized by: increased expression of major histocompatibility complex (MHC) molecules, needed to present antigens to T cells; increased expression of co-stimulatory molecules, such as CD80 and CD86, to promote DC interaction with T cells;[7] and secretion of pro-inflammatory cytokines, such as the Th1-inducing cytokine interleukin (IL)-12, required for anti-tumor activity.[8] Typically, endogenous antigens are presented through MHC-I molecules to CD8^+^ cytotoxic T cells, after proteasomal degradation, while exogenous antigens are classically presented *via* MHC-II to CD4^+^ helper T cells, *via* the endolysosomal route.[2] Notably, DCs are endowed with a unique antigen processing pathway that enables exogenous antigens to undergo proteasomal degradation and be presented in the context of MHC-I molecules to CD8^+^ cytotoxic T cells, in a process called cross-presentation.[9, 10] Furthermore, high affinity peptides bind directly to MHC-I, without previous endocytosis and processing.[11] Since most tumor-associated antigens used for DC vaccination are of exogenous origin, strategies that foment antigen presentation through MHC-I are crucial for the generation of CD8^+^ cytotoxic T cell response, and thereby, for the generation of an anti-tumor immune response.[10, 12, 13]

Glycans that decorate cell membrane proteins play a key role in activating or inhibiting cell-cell and receptor-ligand interactions in several immunological processes.[14–16] The terminal positions of these glycans are typically decorated by negatively charged and non-reducing monosaccharides, named sialic acids.[17] Due to this exposed location, sialic acids are involved in many immunological functions. This includes inhibition of immune cell function through Siglecs recognition,[18] decrease of cellular interactions by providing negative charge repulsion between cells, and masking or impairing recognition by galectin receptors.[19, 20] However, sialic acid content at cell surface is tightly regulated, differing among cell types and varying markedly with regard to cell differentiation and stimuli.[21, 22]

In previous studies, we demonstrated that the cell surface of human monocyte-derived DCs (MoDCs) is highly sialylated and the removal of these sialic acids by sialidases induces DC maturation.[22] Consistently, desialylated MoDCs loaded with toxoid antigen present higher ability to activate autologous T cells.[23] Therefore, our observations suggest that sialic acids present at cell surface of DCs contribute to dampen DC maturation and to down-regulate DCs' ability to activate T cells. Yet, the role of cell surface sialic acids in DC immunogenicity against tumor cells is unknown.

Here, we first sought to investigate whether DCs generated by well-established methods employed in clinical trials (e.g., NCT01042366, NCT00683241, and NCT00442754) have improved anti-tumor immune responses when treated with sialidase. Our data show that sialidase treatment of human MoDCs, loaded with whole tumor cell antigens, improves their ability to specifically activate autologous T cells and consequently, enhances T cells' ability to kill tumor cells. We observed that desialylated MoDCs show increased antigen-presenting and co-stimulatory abilities, resulting in higher polarization of T cells towards a Th1 phenotype. Murine splenic DCs (sDCs) also show similar improved capacity to induce anti-tumor immune response when treated with sialidase. Notably, both MoDCs and sDCs showed increased cross-presentation of antigens through MHC-I to CD8^+^ T cells. These results indicate that desialylation of DCs represents an efficient and powerful tool to elicit tumor-specific immune response, with potential impact in DC-based anti-tumor immunotherapy.

## RESULTS

### Desialylated MoDCs loaded with whole tumor antigens have higher ability to induce anti-tumor T cell responses

**Figure 1:**
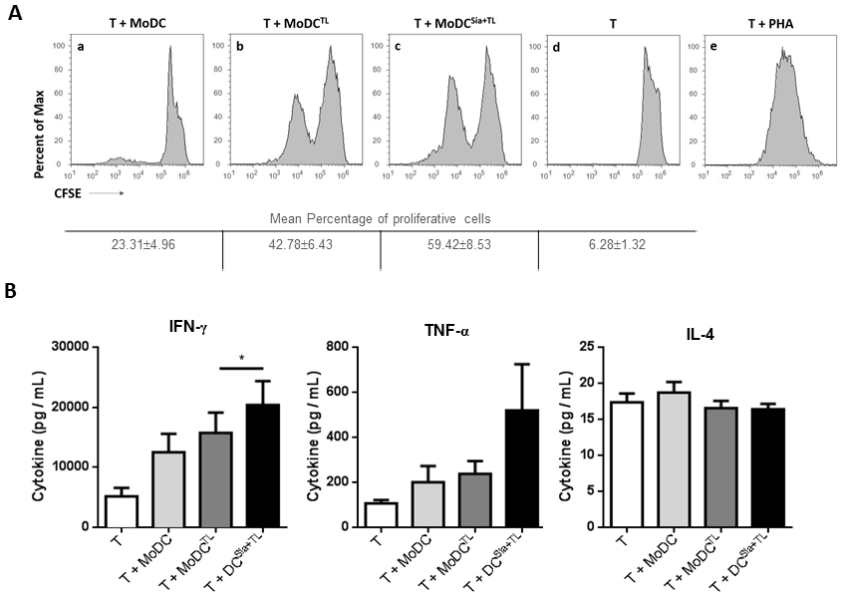
Desialylation of human MoDCs loaded with whole tumor cell antigens induces T cell activation MoDCs were first treated with sialidase (Sia), for 1 hour at 37°C or left untreated, followed by loading with MCF-7 lysates (TL), as source of whole tumor cell antigens. MoDCs were then used in co-cultures with autologous T cells for 4-8 days in the presence of of IL-2. **A.** *Desialylated MoDCs show a higher induction of T cell proliferation*. T cells were previously labeled with CFSE and the progeny, i.e., the percentage of T cells that proliferated, was estimated by flow cytometry, based on the CFSE dilution. The histograms show representative experiments where T cells were co-cultured with: unloaded MoDCs (panel a), MoDCs loaded with MCF-7 lysates (panel b) and sialidase treated MoDCs loaded with MCF-7 lysates (panel c). Unstimulated T cells (panel d) served as negative control and phytohaemagglutinin (PHA)-stimulated T cells (panel e) as positive control. Tabled values represent the mean percentage of proliferative cells ± SEM of 4 independent measurements. **B.** *T cells primed with desialylated MoDCs show higher secretion of Th1 cytokines*. The cytokines secreted into the co-culture supernatants, following T cell stimulation with MoDCs were measured by ELISA (*n* = 7). Graph values represent the concentration (pg/mL) (average ± SEM). Statistically significant differences were calculated using *t*-test (*P < 0.05).

We first sought to assess the effect of sialic acid removal from the cell surface of clinical relevant DCs on their ability to induce specific anti-tumor T cell responses. Therefore, we primed T cells with autologous desialylated MoDCs loaded with MCF-7-tumor cell lysates. Based on CFSE dilution method, T cells primed with desialylated MoDCs showed higher proliferation, when compared to those primed with fully sialylated MoDCs (Figure 1A). To further characterize the profile of T cells generated by desialylated MoDCs stimuli, the secretion of the Th1 cytokines - TNF-α and IFN-γ - and of the Th2 cytokine - IL-4 - was analysed. As shown in Figure 1B, when compared with T cells primed by fully sialylated MoDCs, T cells primed by desialylated MoDCs secreted significantly higher levels of IFN-γ. However, the expression of IL-4 by T cells was not altered when primed either by fully sialylated or desialylated MoDCs (Figure 1B). Thus, desialylated MoDCs have improved capacity to prime autologous T cells, as shown by their ability to induce T cell proliferation and production of Th1 cytokines, which are crucial for the induction of anti-tumor immune responses.

**Figure 2:**
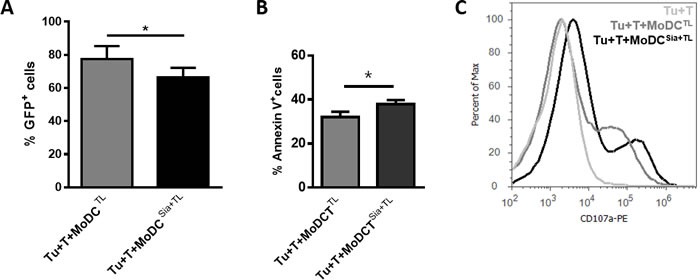
Desialylation of human MoDCs loaded with whole tumor cell antigens improves T cell cytotoxicity against tumor cells MoDCs obtained from HLA-A*02:01 donors were treated with sialidase (Sia), for 1 hour at 37°C or left untreated, and loaded with lysates of the breast cancer cell line MCF-7 (TL). Autologous CD3^+^ T cells (T) were co-cultured with MoDCs loaded with MCF-7 lysates (MoDC^TL^), or MoDCs that were sialidase treated and loaded with MCF-7 lysates (MoDC^sia+TL^). After 3 weeks, CD3^+^ T cells were co-cultured for 5 hours with MCF-7 cells (Tu) in a ratio of 1 Tu: 10 T cells; non-stimulated T cells were cultured with MCF-7 as control. (**A.**, **B.)** *T cells primed with desialylated human MoDCs loaded with MCF-7 cell lysates induce higher tumor cell apoptosis*. Tumor cell death was evaluated by flow cytometry by assessing both the intensity of GFP reporter (**A)** and Annexin V and 7-AAD reactivity. (**B)** Values represent the percentage ± SEM of viable tumor cells based on the maintenance of GFP fluorescence (GFP^+^) in 4 independent assays (graph A) and the percentage ± SEM of apoptotic tumor cells assessed by staining with Annexin-V and 7-AAD, in 3 independent measurements (graph B). Statistically significant differences were calculated using *t*-test (*P < 0.05). **C.** *T cells primed with desialylated MoDCs show higher degranulation.* The degranulation of cytotoxic T cells against tumor cells was determined based on the CD107a expression. Representative Histogram is a representative experiment of 4 independent assays.

The cytotoxicity of primed HLA-A*02:01^+^ T cells was then tested against the MCF-7 breast cancer cells stably expressing GFP (MCF-7-GFP). As shown in Figure 2A and 2B, tumor cells become significantly more apoptotic when incubated with T cells that had been previously primed with desialylated MoDCs loaded with tumor-antigens, compared to those incubated with T cells primed with fully sialylated MoDCs. Concomitantly, the highest degranulation of T cells, was observed when these were primed by desialylated MoDCs prior contact with tumor cells, as shown by the expression of cell surface CD107a (Figure 2C).

### Desialylated human MoDCs loaded with tumor antigens show higher levels of maturation and co-stimulatory markers and IL-12 secretion

**Figure 3:**
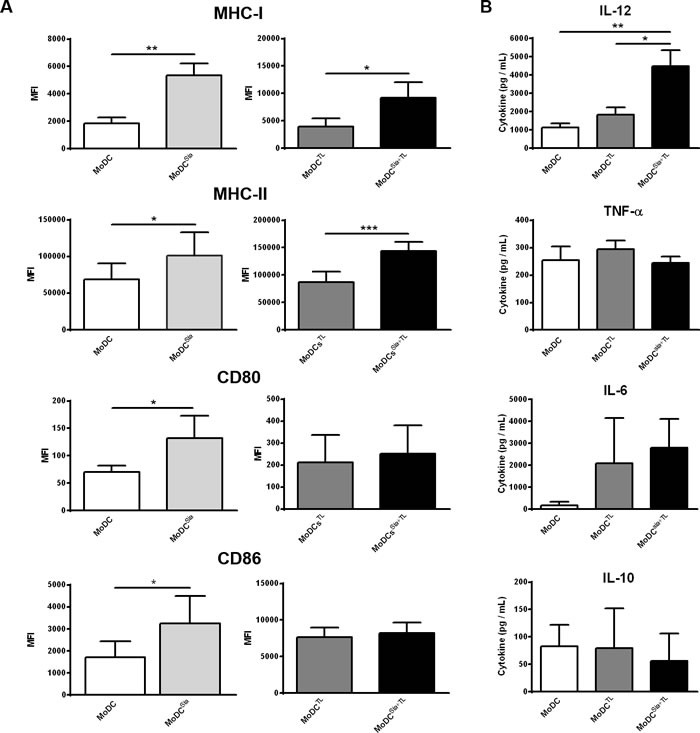
Desialylated MoDCs show higher levels of maturation and expression of pro-inflammatory cytokines **A.** *Desialylated MoDCs show a higher maturation phenotype than fully sialylated MoDCs.* The expression of several maturation markers was evaluated by flow cytometry. MoDCs were first treated with sialidase (Sia) for 1h at 37°C and then loaded (A, right panel) or not (A, left panel) with MCF-7 lysates (TL), for 3h at 37°C, as described in Materials and methods. Graph values represent the mean fluorescence intensity (MFI) (average ± SEM) of at least 3 independent assays. Statistically significant differences were calculated using *t*-test (*P < 0.05, **P < 0.01 and ***P < 0.0001) and refers to the difference between untreated and sialidase treated conditions. **B.** *Sialidase treatment of MoDCs elicits IL-12 secretion.* The cytokines secreted by MoDCs following sialidase treatment were measured by ELISA. Data represent the cytokine concentration (pg/mL) (average ± SEM) of 6 independent experiments. Statistically significant differences were calculated using *t*-test (*P < 0.05 and **P < 0.01) and refers to the difference between untreated and sialidase treated conditions.

To shed light on the mechanism behind the observed higher anti-tumor T cell responses induced by desialylation of MoDCs, we analysed the maturation profile of desialylated MoDCs, loaded or not with tumor-antigens. To do this, the expression of MHC-I and MHC-II, and the co-stimulatory molecules CD80 and CD86 were assessed after sialidase treatment. Sialidase treatment of the unloaded MoDCs increased significantly the expression of all maturation markers compared to untreated MoDCs (Figure 3A, left panels). Similarly, a significantly improved maturation phenotype was observed after sialidase treatment of MoDCs loaded with MCF-7 lysates (Figure 3A, right panels). Yet, sialidase effect was restricted to MHC-I and MHC-II molecules, and did not extent to the co-stimulatory molecules CD80 and CD86 (Figure 3A, right panel). The combination of sialidase treatment with tumor-antigen upload did not alter the expression of other co-stimulatory molecules, such as CD40, and of the chemokine receptor CCR7, when compared with fully sialylated MoDCs (results not shown). Given the Th1 profile of T cells primed by desialylated MoDCs, we further evaluated the effect of sialic acid removal from DC surface on their ability to secrete Th1-inducing cytokines, IL-12, TNF-α and IL-6, and the immune-inhibitory Th2-inducing cytokine IL-10. A significant increase in IL-12 secretion was observed in desialylated MoDCs loaded with tumor antigens compared with those that were not treated with sialidase (Figure 3B). Although not statistically significant, the production of IL-10 was slightly reduced, in agreement with a Th1 phenotype. The expression of TNF-α and IL-6 was not altered. Our data show that desialylation of MoDCs boosts DC maturation, in particular the expression of MHC-I and MHC-II, and secretion of the Th1-inducing cytokine IL-12

### Desialylated murine sDCs show higher levels of maturation and co-stimulatory markers

**Figure 4:**
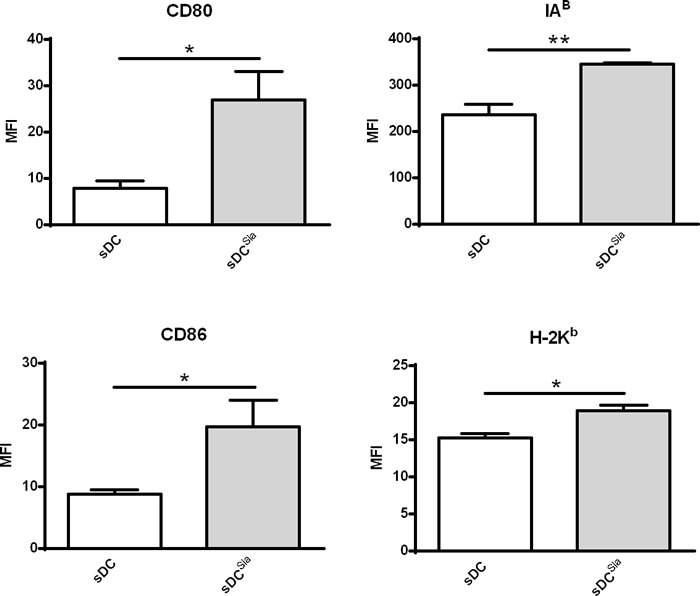
Sialidase treatment of murine sDCs induces their maturation Murine sDCs were treated with sialidase or left untreated and the maturation markers evaluated by flow cytometry. Statistical significance (*P < 0.05 or **P < 0.001) refers to the difference between fully sialylated and desialylated sDCs, calculated using *t*-test. Values represent MFI (average ± SEM) of at least 6 independent assays.

To investigate whether the sialidase effect was restricted to human DCs, we analysed the maturation profile of desialylated murine sDCs. Therefore, we analysed the expression of MHC and co-stimulatory molecules by sDCs after sialidase treatment. sDCs that were treated with sialidase showed significantly higher expression of H-2k^b^ (MHC-I), IA^b^ (MHC-II), CD80 and CD86 molecules (Figure 4). Thus, desialylation of murine sDCs leads to a stronger induction of maturation, similar to human MoDCs.

### Desialylation of murine sDCs induces antigen-specific CD4^+^ and CD8^+^ T cell proliferation and activation

**Figure 5:**
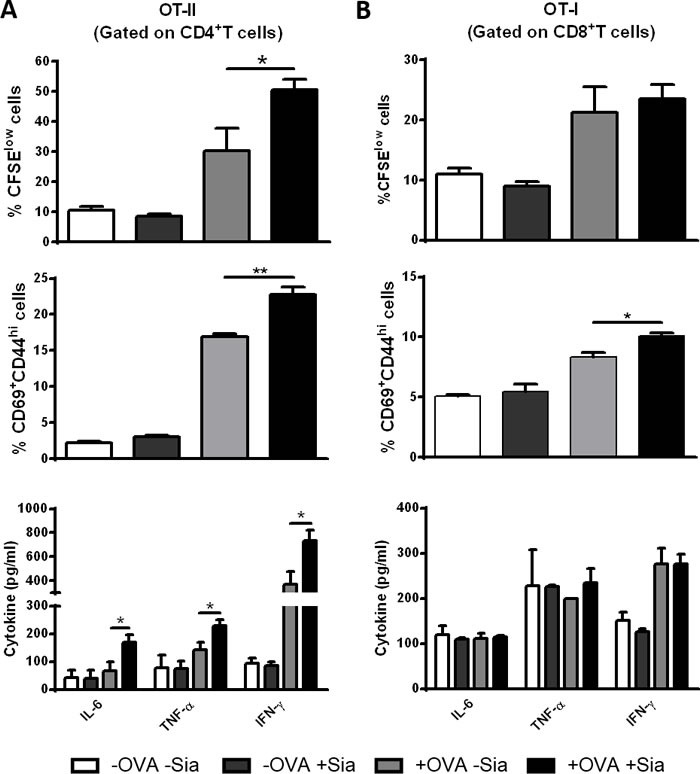
Sialidase treatment of murine sDCs induces OVA-specific CD4^**+**^ and CD8^**+**^ T cell proliferation and activation Murine sDCs were pulsed with OVA for 4h and treated or not with sialidase for 1h. **A.** *Desialylated sDCs strongly induce proliferation, activation and differentiation of CD4* + *T cells into a Th1 effector phenotype.* Splenocytes from OT-II mice were isolated and co-cultured with sDCs for 72h in a ratio of 1:2 (DCs:splenocytes). After 72 h, the percentage of proliferating and activated CD4^+^ T cells was determined by flow cytometry using the CFSE dilution assay and evaluating the expression of CD69 and CD44, respectively (gating on CD4^+^ T cells). **B.** *Desialylated sDCs induce activation of CD8* + *T cells.* Splenocytes from OT-I mice were isolated and co-cultured with sDCs for 72h in a ratio of 1:2 (sDCs:splenocytes) The proliferation of CD8^+^ T cells was determined by CFSE dilution and the activation determined based on the expression of CD69 and CD44 (gating on CD8^+^ T cell). The secretion of IFN-γ, IL-6, and TNF-α into the supernatants of co-cultures was evaluated by ELISA. Graph values represent the percentage of CFSE^low^ cells, percentage of CD69^+^CD44^high^ cells and cytokine concentration (pg/mL) (average ± SEM) from 3 independent assays. Statistical significance (*P < 0.05 or **P < 0.001), calculated using *t*-test, refers to the difference between fully sialylated and desialylated sDCs.

Since desialylated sDCs showed an increased maturation phenotype, we next examined whether desialylation would result in increased ability of sDCs to prime antigen-specific T cells. To assess this, sDCs were loaded with the OVA model antigen, desialylated and then co-cultured either with OVA-specific CD4^+^ or CD8^+^ T cells, from transgenic OT-II or OT-I mice, respectively. T cell proliferation, expression of activation markers and cytokine secretion by T cells, were assessed. Compared with fully sialylated OVA-pulsed sDCs, desialylated OVA-pulsed sDCs induced significantly higher OVA-specific CD4^+^ T cell proliferation (Figure 5A). Accordingly, the activation of CD4^+^ T cells was significantly improved when these cells were primed with desialylated OVA-pulsed sDCs, as demonstrated by the evaluation of the percentage of CD69^+^CD44^high^ T cells. To assess whether priming with desialylated sDCs would induce a Th1 profile on CD4^+^ T cells, we analysed the levels of IL-6, TNF-α and IFN-γ secretion by primed CD4^+^ T cells. As shown in Figure 5A, the secretion of all three Th1-cytokines was significantly increased when CD4^+^T cells were primed with desialylated OVA-pulsed sDCs compared to T cells primed with fully sialylated OVA-pulsed sDCs. Taken together, these results show that sialidase treatment of sDCs significantly improves their ability to induce the proliferation and activation of CD4^+^ T cells with improved Th1 profile.

Regarding CD8^+^ T cells, desialylated sDCs showed a modest tendency to induce proliferation, but a considerable ability to activate them, based on the percentage of CD69^+^CD44^high^ T cells (Figure 5B). The secretion of IL-6, TNF-α and IFN-γ cytokines was not altered when CD8^+^ T cells were primed by desialylated sDCs (Figure 5B).

### Desialylation of murine splenic DCs leads to improved T cell-mediated tumor cell death

**Figure 6:**
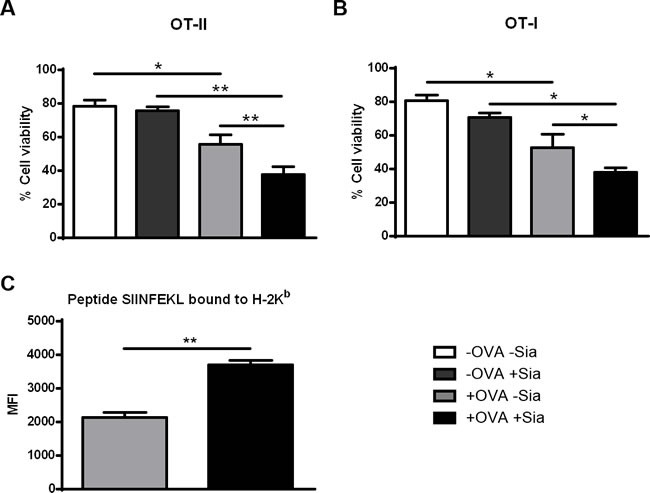
Sialidase treatment of murine sDCs loaded with OVA improves T cell cytotoxicity against tumor cells Induction of cytolysis of tumor cells was tested by co-culturing untreated (grey light bars) or sialidase treated (black bars) OVA-pulsed DCs and splenocytes from either OT-II (**A.)** or OT-I (**B.)** splenocytes for 72h. After, primed T cells and OVA-expressing B16 mouse melanoma cells (B16-OVA) were co-cultured for 24h. B16-OVA tumor cell death was assessed by staining with Annexin-V and 7-AAD and analysed by flow cytometry. Graph values represent the percentage ± SEM of viable tumor cells of 2 independent assays. Statistical significance (*P < 0.05 or **P < 0.001) refers to the difference between fully sialylated and desialylated sDCs (*n* = 2). (**C.**) *Desialylated sDCs show improved presentation of endocytosed ovalbumin-derived peptide SIINFEKL, bound to MHC class I*. Murine splenic DCs were first incubated with OVA and treated (black bars) or not (grey bars) with sialidase. After these treatments, the presentation of the ovalbumin-derived peptide SIINFEKL bound to H-2K^b^ (murine MHC class I) epitopes was analysed by flow cytometry, using the 25-D1.16 monoclonal mAb. Graphs show the MFI ± SEM of 2 independent assays. Statistical significance (**P < 0.001) refers to the difference between fully sialylated and desialylated sDCs and it was determined using *t*-test.

To investigate whether OVA-specific CD4^+^ and CD8^+^ T cells primed with desialylated sDCs would have a higher ability to induce tumor cell cytotoxicity, we co-cultured the primed T cells with the OVA-expressing melanoma cell line B16-OVA. Notably, B16-OVA tumor cells that were incubated either with CD4^+^ (Figure 6A) or CD8^+^ T cells (Figure 6B), previously primed with desialylated OVA-pulsed sDCs, showed significantly less viability than those incubated with T cells primed with fully sialylated OVA-pulsed sDCs (Figure 6). Thus, desialylation of sDCs improves their ability to prime antigen-specific T cell-mediated immune response against tumor cells.

### Desialylated murine sDCs show improved presentation of OVA-derived peptide

Antigen presentation by DCs to cytotoxic T cells includes cross-presentation of antigens, which is an essential mechanism to present endocytosed exogenous antigens *via* MHC-I to CD8^+^T cells and therefore, to induce anti-tumor immunity.[9]

Since activation of CD8^+^ T cells by OVA-pulsed sDCs implies cross-presentation of peptides derived from the endocytosed OVA, we assessed the ability of sDCs to cross-present OVA-derived peptides through MHC-I. For this purpose, OVA-pulsed sDCs treated or not with sialidase, were stained with the 25-D1.16 mAb that specifically recognizes the processed OVA peptide- SIINFEKL bound to H2-K^b^. This assay readily detected cross-presenting murine sDCs and showed that, sialidase treated sDCs presented significant higher cross-presentation of the SIINFEKL peptide through MHC-I (Figure 6C). Thus, the data indicates that desialylation of murine sDCs improves the antigen cross-presentation *via* MHC-I.

### Sialidase treatment of human MoDCs improves antigen presentation *via* MHC-I

We next determined whether improved antigen cross-presentation was also extensible to human desialylated DCs. Additionally, we also analysed whether DC desialylation would affect the presentation of exogenously supplied high affinity peptides that bind directly to MHC-I molecules, without previous cell internalization.

**Figure 7:**
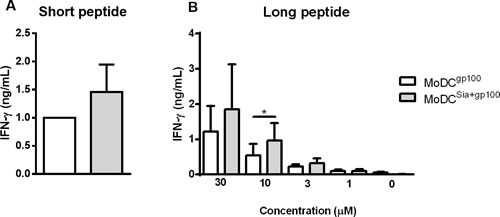
Sialidase treatment of human MoDCs improves antigen cross-presentation *via* MHC-I to CD8^**+**^ T cells Gp100-specific CD8^+^ T cells were co-cultured overnight with gp100-loaded MoDCs, that have been previously treated or not with sialidase (MoDC^sia+gp100^ and MoDC^gp100^, respectively). To evaluate the activation of CD8^+^ T cells, the secretion of IFN-γ cytokine was measured by ELISA. **A.** Short gp100 peptides or **B.** different concentrations of the long gp100 peptide were used to load MoDCs. Graph values are normalized to the condition of MoDC^gp100^ with the short peptide. Data refers to the IFN-γ secretion (ng/mL) (average ± SEM) of 3 independent experiments. Statistical significance (*P < 0.05) refers to the difference between fully sialylated and desialylated gp100-loaded MoDCs.

To address this, we loaded HLA-A*02:01^+^ MoDCs with short and long gp100 peptides that share the gp100_280-288_ sequence (YLEPGPVTA) that binds to HLA-A*02:01 and tested their ability to prime gp100_280-288_ specific CD8^+^ T cell clones. As shown in Figure 7, desialylated MoDCs loaded either with gp100 short (Figure 7A) or long peptide (Figure 7B) showed higher ability to stimulate CD8^+^ T cells. The data suggests that desialylation of human MoDCs improves antigen presentation *via* MHC-I. This is due to a significant increase in antigen cross-presentation and to the fact that exogenously supplied peptides bind to desialylated MHC-I with higher affinity.

## DISCUSSION

The successful use of DCs as a cancer vaccine has been limited and only a small percentage of objective clinical responses have been observed in clinical trials.[4, 29] The immediate hurdle for the success of DC immunotherapy is to improve their ability to prime T cells, so they can elicit an effective and long-lasting anti-tumor immune response.

Sialylation of cell surface glycans may stimulate or inhibit immune cell recognition, differentiation and maturation. We were the first to report that human MoDCs, currently employed in clinical trials, display a high content of cell surface α(2,6)- and α(2,3)- sialylation.[22] Whilst matured MoDCs show decreased sialylation,[23, 30] MoDCs with higher sialylation are more tolerogenic and immature.[31] Also, sialic acid removal or deficiency improves DCs' maturation,[23, 32] suggesting that sialylation dampens DC activity. Yet, it was unknown whether shortage of sialic acid improves the ability of MoDCs to induce proper anti-tumor immune responses. Accordingly, in this study we assessed the anti-tumor immunity of desialylated MoDCs. To address this, MoDCs were desialylated using a recombinant sialidase, which rapidly leads to a transient cell surface desialylation, while preserving MoDCs' viability. Importantly, desialylation was observed up to 24h after treatment and typical sialic acid content of MoDCs was fully recovered after 48h (data not shown).

Our results showed that human desialylated MoDCs, pulsed with whole tumor antigens from the breast cancer cell line MCF-7, induced a significant higher proliferation of T cells, compared to fully sialylated MoDCs. Moreover, increased T cell proliferation was accompanied by increased secretion of IFN-γ, typical of a Th1-induced response. IFN-γ is a pleiotropic cytokine whose biological role has been extensibly associated with pro-cytotastic and pro-apoptotic effects against several malignancies.[33] This powerful cytokine up-regulates MHC-I molecule expression on tumor cells, increasing tumor antigen presentation,[34, 35] and participates in the recruitment and activation of several immune cells.[36] Concomitantly, these T cells primed by desialylated MoDCs exhibited the highest cytotoxic activity against tumor cells. This higher capacity of desialylated antigen-loaded MoDCs to prime T cells may also be explained by the increased expression of antigen-presenting and co-stimulatory molecules, which is in agreement with our previous reports.[23] Furthermore, desialylation of MoDCs loaded with tumor cell lysates resulted in a remarkable higher secretion of the IL-12 cytokine. This cytokine triggers the differentiation of CD4^+^ T cells into IFN-γ producing cells, thus inducing Th1 responses. In addition, the secretion of IL-10 was also modestly reduced, enforcing a Th1 profile, since IL-10 blocks Th1 differentiation[37, 38] and limits maturation of DCs by down-regulating co-stimulatory molecules and IL-12 production.[39, 40] Nevertheless, it was observed some variability in cytokine secretion by MoDCs, which can be explained by the fact that these generated human DCs are characterized by inter- and intra-donor variations.[41] However, and in spite of donor-to-donor variability, sialidase treatment induces a significant IL-12^high^ DC phenotype, vital to T cell differentiation into Th1 effectors and to the generation of anti-tumor responses. Although increased maturation of desialylated MoDCs had been previously described,[23, 32] our data are the first to indicate that desialylated MoDCs loaded with tumor antigens have higher capacity to prime T cell-mediated anti-tumor immune responses and that is probably due to an increased maturation state.

Notably, experiments with murine sDCs are the first to show that, as in human, desialylation of murine sDCs, improves the expression of MHC-I/-II presenting molecules and of CD80 and CD86 co-stimulatory molecules. Moreover, desialylated sDCs pulsed with OVA-antigen showed remarkable higher ability to boost proliferation and activation of OVA-specific CD4^+^ T cells, as well as secretion of IFN-γ, IL-6 and TNF-α. Notably, both CD4^+^ and CD8^+^ OVA-specific T cells primed by desialylated OVA-pulsed DCs, exhibited higher cytotoxic activity towards OVA-expressing tumor cells, when compared to those primed with fully sialylated OVA-pulsed sDCs.

Importantly, our data shows for the first time that sialidase treatment of human or murine DCs improves their antigen cross-presentation ability. Sialidase treatment of murine sDCs improved presentation of endocytosed ovalbumin-derived peptide SIINFEKL, bound to MHC-I. Commensurably, sialidase treated HLA-A*02:01 MoDCs pulsed either with the long or short gp100 peptide displayed enhanced antigen presentation through MHC-I, and consequent activation of gp100_280-288_ specific CD8^+^ cytotoxic T cells. Since the long peptide needs to be internalized, exported from lysosome to the cytosol and then conducted into the cross-presentation process, our data is suggestive that desialylation of human DCs improves their antigen cross-presentation. It is possible that DC desialylation stimulates critical receptors that favor antigen export into the cytosol, such as the mannose receptor.[10] Additionally, data obtained with the short gp100 peptide, suggests that desialylation also improves direct binding of exogenous peptides to cell surface MHC-I. Noteworthy, our data also suggests that increased expression of MHC-I (either due to recycling, or newly synthesis) is a possible mechanism behind improved antigen presentation through MHC-I. Interestingly, a previous study reported that desialylation of CD8^+^ T cells is required and adequate for T cell effective anti-tumor response against glioma cancer.[42] Thus, future studies at molecular level are envisaged to better understand how sialidase treatment improves anti-tumor mechanisms.

Collectively, our data show that sialidase treatment of DCs is able to induce all the phenotypical and functional features of maturation, including increased antigen presentation, co-stimulatory molecules and secretion of Th1 cytokines. More important, this is the first report showing that an enzymatic treatment through extrinsic desialylation of *in vitro* generated DCs elicits better presentation of tumor antigens through MHC-I. In agreement, we show that removal of sialic acid considerably boosts DC anti-tumor immune response. Therefore, our findings suggest that sialidase treatment can be used as a powerful tool to improve the effectiveness of therapeutic cancer vaccines.

## MATERIALS AND METHODS

### Reagents

Culture media, RPMI-1640, DMEM and IMDM, fetal bovine serum (FBS), non-essential amino acids, sodium pyruvate, trypsin-EDTA, L-glutamine and GlutaMAX were purchased from Gibco (Grand Island, NY, USA). Recombinant human granulocyte-macrophage colony-stimulating factor (GM-CSF), IL-2, tumor necrosis factor (TNF)-α, and the cell isolation immunomagnetic beads were obtained from Miltenyi Biotec (Bergisch Gladbach, Germany). Recombinant human IL-4 was purchased from R&D Systems (Minneapolis, MN, USA). The CellTrace™ CFSE Cell Proliferation Kit was purchased from Molecular Probes, and Annexin V from Immunotools (Friesoythe, Germany).

The following anti-human antibodies (mAbs) were used: fluorescein isothiocyanate (FITC)-conjugated anti-CD1a from BD Biosciences (San Jose, CA, USA); phycoerythrin (PE)-conjugated anti-CD1c from BioLegend (San Diego, CA, USA); allophycocyanin (APC)-conjugated anti-HLA-DR from Immunostep (Salamanca, Spain); and APC conjugated anti-CD3, anti-CD4; peridinin chlorophyll protein (PercP)-conjugated anti-CD8; PE-conjugated anti-CD14, anti-CD40, anti-CD69 and anti-CD80; and FITC-conjugated anti-CD86 mAbs from Immunotools.

The following anti-mouse mAbs were used: FITC-conjugated anti-CD11c, APC-conjugated anti-B220, anti-CD11b, PercP-conjugated anti-CD8α, PE-conjugated anti-IA^b^ and anti-CD86 mAbs from BD Biosciences; PercP-conjugated anti-CD4, FITC-conjugated anti-H-2K^b^, biotin-conjugated CD80 and PE-conjugated anti-SIINFELK/H-2Kb mAbs from eBioscience (San Diego, USA); PE-conjugated streptavidin (Biolegend) was used in combination with biotin-conjugated anti-CD80 mAb. Unless otherwise stated, remaining reagents and media were purchased from Sigma Chemical Co. (Sigma, St Louis, MO, USA).

### Flow cytometry

Cell surface staining was performed using fluorescence-conjugated or unlabeled mAb followed by fluorescently-conjugated secondary mAb, according to manufacturer instructions. Data was acquired using either an Attune Acoustic Focusing Cytometer (Applied Biosystems, USA) or a FACSCalibur Cytometer (Becton-Dickinson, FranklinLakes, NJ, USA) and analysed using either the Attune Cytometric Software v2.1 or the FlowJo software version 10.0.5 (TreeStar, San Carlos, CA, USA).

### Human tumor cell line culture

The HLA-A*02:01 positive breast cancer cell line MCF-7 was obtained from American Type Culture Collection and the MCF-7-GFP variant (constitutively expressing the green fluorescent protein (GFP)) from Cell Biolabs, Inc. (cat. Number AKR-211). Cells were routinely cultured in DMEM media with 10% FBS, 2 mM L-glutamine, and 100 μg/mL penicillin/streptomycin.

### Generation of human MoDCs

Peripheral blood mononuclear cells (PBMCs) were isolated from buffy coats of human peripheral blood from healthy volunteers provided by the Portuguese Blood and Transplantation Institute, after informed consent and approval by institutional ethical committee. Monocytes were enriched using anti-CD14 mAb-coated immunomagnetic beads and cultured, as described previously.[24]

### Mice

Six to eight weeks old C57BL/6 (B6) mice, and the B6 transgenic mice OT-I (B6.OT-I; producing OVA-specific MHC-I restricted CD8^+^ T cells) and OT-II (B6.OT-II; producing OVA-specific MHC-II restricted CD4^+^ T cells) were housed and bred at the animal facility of Instituto Gulbenkian de Ciência (IGC, Oeiras, Portugal). Mice were sacrificed by carbon dioxide inhalation followed by cervical dislocation. All procedures were approved by the Institutional Animal Care and Use Committee (IACUC), in agreement with the Federation of European Laboratory Animal Science Associations (FELASA) directives, approval ID number AO10/2010.

### Murine cell culture

The mouse melanoma cell line B16, transfected with the FMS-like tyrosine kinase 3 ligand gene (B16-Flt3-L) was gently provided by Dr. J. Lafaille (Skirball Institute of Biomolecular Medicine, NYU, USA) and cultured in complete RPMI medium. The B16 cell line transfected with the ovalbumin gene (B16-OVA) was gently provided by Dr. L. F. Moita (IMM, Lisboa, Portugal) and was grown in complete DMEM medium. Cells were split approximately every three days, after cell detachment using trypsin/EDTA.

### Generation of murine DCs

To generate large numbers of sDCs, female C57BL/6 mice were injected subcutaneously in the flank with 2 × 10^5^ B16-Flt3-L cells, as described.[25] After 10 to 15 days, mice were sacrificed and splenocytes isolated by mechanical disruption of spleens. sDCs were further sorted by positive immunomagnetic selection using anti-CD11c mAb-coated immunomagnetic beads following manufacturer's instructions. The isolated cell fraction was typically ~ 97% CD11c^+^ cells. Further characterization of DC subsets was performed (Supplementary Figure S1).

### T cell isolation and culture

The human T cells used in this work comprised the CD14^−^ cell fraction of PBMCs (> 70% CD3^+^ cells), obtained after immunomagnetic isolation of monocytes (CD14^+^), described above. For cytotoxicity experiments, T cells were further enriched using anti-CD3 mAb-coated immunomagnetic beads (> 97% CD3^+^ cells). The CD8^+^ T cells transduced with a TCR gene specific for the HLA-A*02:01 restricted gp100_280-288_ peptide were cultured as described.[26] Murine T cells were obtained from splenocytes from B6.OT-I and B6.OT-II TCR transgenic mice, obtained by spleen mechanical disruption, followed by red blood cell lysis.

### Antigen loading and stimulation of human and murine DCs

Lysates of tumor cells were prepared through freeze (−80°C) and thaw (37°C) cycles and the cell debris removed by centrifugation. Complete cell lysis was confirmed microscopically by trypan blue exclusion and by culture test. The protein concentration of the tumor cell lysates was determined using Pierce BCA Protein Assay kit (Thermo Scientific; Germany). MoDCs were incubated with tumor cell lysate (1mg per 5×10^6^ MoDCs, per mL), in complete RPMI medium, for 4h at 37°C. When needed, MoDCs were stimulated for 24h with TNF-α (1000 IU/mL).

Murine sDCs were incubated with OVA (0.1mg per 0.5×10^6^ sDCs, per mL), in complete RPMI medium, for 4-6h at 37°C.

### Sialidase treatment

Cells were treated with 0.1 U/mL of sialidase from *Clostridium perfringens* (Roche Diagnostics, Basel, Switzerland) for 60 min at 37°C. Sialic acid removal was confirmed by lectin staining with FITC-labeled *Sambucus nigra* lectin (SNA), -*Maackia amurensis* lectin II (MAA) and -*Peanut agglutinin lectin* (PNA) (Vector Laboratories, Peterborough, UK) (Supplementary Figure S2). Control experiments were performed without sialidase or with heat-inactivated sialidase (20 min at 100°C). After treatment, cell viability was evaluated by staining with 7-Aminoactinomycin D (7-AAD, BD Biosciences) and Annexin V and analysed by flow cytometry (Supplementary Figure S3).

### Proliferation assays

T cell proliferation was assessed based on the carboxyfluorescein succinimidyl ester (CFSE) dilution assay by flow cytometry. Briefly, T cells were labeled with CFSE, according to manufacturer instructions and co-cultured with DCs in complete RPMI at 37°C. For experiments using human cells, the ratio used of MoDC:T cells was 1:5. Experiments using murine cells, sDCs were cultured with splenocytes from OT-I or OT-II mice in a ratio of 1:2, for 72h.

### Antigen presentation assay

To evaluate antigen presentation, HLA-A*02:01 MoDCs were loaded with gp100 antigen peptides: gp100_280-288_ short peptide (YLEPGPVTA) and gp100_280-304_ long peptide (YLEPGPVTANRQLYPEWTEAQRLDC) that contain an amino acid sequence (underlined) that binds to the HLA-A*02:01 allele. MoDCs were incubated with several concentrations of gp100 peptides in RPMI medium, for 3 to 4 hours, at 37°C. MoDCs were then co-cultured with gp100-specific CD8^+^ T cells,[26] in IMDM medium, in a MoDC: T cell ratio of 3: 10. Following overnight incubation, the secretion of IFN-γ cytokine into supernatant was measured by Enzyme-linked immunosorbent assay (ELISA).

To assess the presentation of the OVA derived peptide SIINFEKL bound to H-2K^b^ (murine MHC-I), sDCs loaded with OVA were stained with the anti-SIINFELK/H-2K^b^ (25-D1.16 clone) mAb and analysed by flow cytometry.

### Cytotoxicity assays

HLA-A*02:01^+^ MoDCs loaded with tumor cell lysates were used to prime autologous CD3^+^ T cells, as described above. Co-cultures were maintained for 2-3 weeks, and T cells were pulsed again after the first week with antigen-loaded HLA-A*02:01^+^ MoDCs. IL-7 (5 ng/mL) was added at day 1 of culture and at the days of MoDC reload; IL-2 (10UI/mL) was added at day two. Primed T cells were cultured against the target tumor cells MCF-7-GFP, in a ratio of 10 T cells per 1 tumor cell, for 5h at 37°C. Cell death of MCF-7-GFP was assessed by flow cytometric analysis of both the reduction of GFP fluorescence intensity, due to the acidification of cell cytoplasm,[27] and Annexin V and 7-ADD staining. T cell cytolytic capacity, i.e., T cell degranulation, was also evaluated by flow cytometric analysis of the cell surface expression of the lysosomal-associated membrane protein 1 (CD107a, LAMP-1), typically expressed in the membrane of cytotoxic granules.[28]

For experiments with murine cells, splenocytes from OT-I and OT-II mice were primed with OVA-loaded sDCs, for 72h, as described above. The mouse melanoma cell line B16-OVA was labeled with CFSE and used as target tumor cell. Primed splenocytes were added to the B16-OVA cells at a ratio of 5 splenocytes per 1 tumor cell and co-cultured for 24h at 37°C. Tumor cell killing was evaluated by flow cytometry, based on Annexin-V and 7-ADD staining of CFSE^+^ cells.

### Cytokine analysis

The cytokine production was measured using ELISA technique, according to the manufacturer's instructions. Human IL-4, IL-6, IL-10, IL-12, TNF-α and IFN-γ ELISA development kits were purchased from Immunotools. Mouse IL-6, TNF-α and IFN-γ ELISA development kits were purchased from Prepotech (Hamburg, Germany). Cytokine concentration was calculated using the specific standard curves.

### Statistical analysis

Data were analysed using GraphPad Prism 6 (GraphPad Software, Inc). The significance of differences between series of results with three or more groups was assessed using the one-way ANOVA. Data with two groups were analysed by Student-*t*-test. Tests were considered statistically significant when *P* < 0.05 (*), *P* < 0.01 (**) and *P* < 0.005 (***).

## SUPPLEMENTARY FIGURES


