# Supplementary material for: Sialic acid removal from dendritic cells improves antigen cross-presentation and boosts anti-tumor immune responses
Source: Oncotarget. 2016 May 17;7(27):41053–66. doi: 10.18632/oncotarget.9419 (PMC5173042; doi:10.18632/oncotarget.9419)
Supplement: Supplementary file 1 [file oncotarget-07-41053-s001.pdf]

Sialic acid removal from dendritic cells improves antigen cross-presentation and boosts anti-tumor immune responses

Supplementary Material

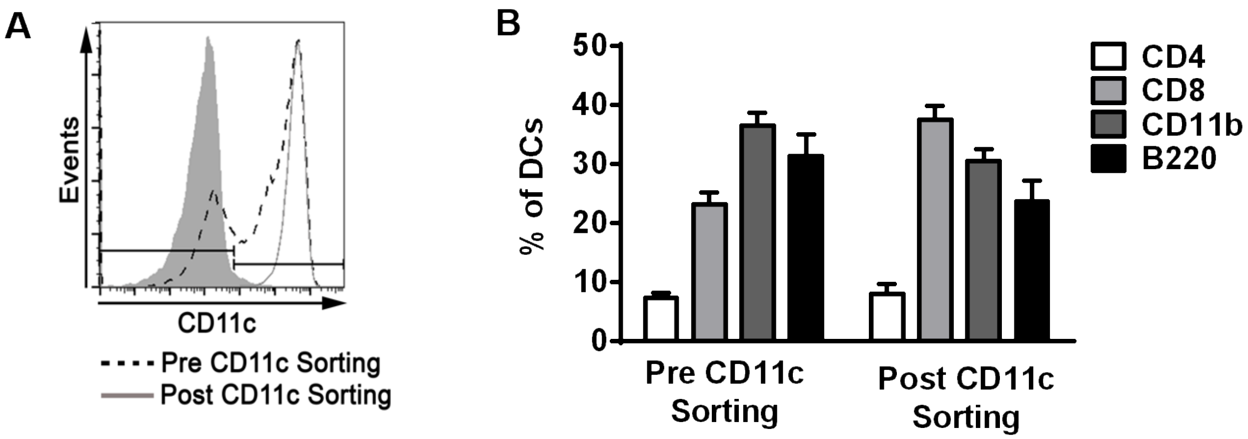

Figure S1. Characterization of splenic DC subsets after CD11c positive selection. (A) *Representative histogram of CD11c cells after positive selection*. After sorting, the purity of the population was typically around 97%. (B) Proportion *of each conventional sDC subset before and after CD11c cell sorting*. In order to assess whether CD11c positive selection would affect the distribution of the different subsets of splenic DCs, we stained CD11c cells with CD4, CD8a (lymphoid DCs), B220 (plasmacytoid DCs) and CD11b (myeloid DCs) markers. After CD11c positive selection, the relative proportions of different sDC subsets remained roughly similar, except for the lymphoid and plasmacytoid populations that respectively increased and decreased slightly.

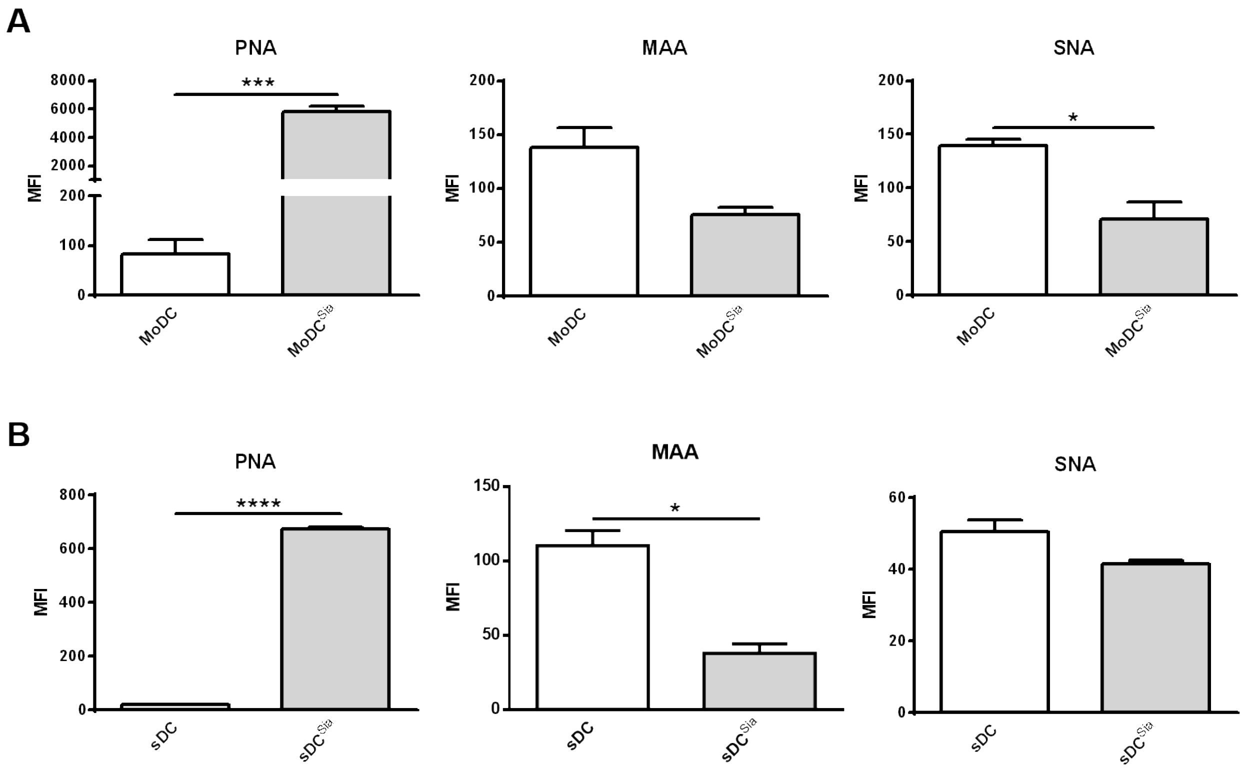

Figure S2. Lectin staining to test the efficacy of sialidase treatment. Human MoDCs (A) and murine sDCs (B) were treated with sialidase (grey bars) or left untreated (white bars) and stained with *Sambucus nigra lectin* (SNA; recognizing α(2,6)-sialic acids), *Maackia amurensis lectin* II (MAA; recognizing α(2,3)-sialic acids) and *Peanut agglutinin lectin* (PNA; recognizing T antigen- Galβ1-3GalNAcα1-Ser/Thr) and analysed by flow cytometry. Values represent the means of the MFI of at least six independent assays. Statistical significance (**P* < 0.05 or ****P* < 0.0001) refers to the difference between untreated and sialidase-treated DCs. Sialidase treatment decreased MAA binding and increased PNA staining of both human MoDCs and murine sDCs, resulting from the removal of α(2,3)-linked sialic acids; removal of α(2,6)-linked sialic acids after sialidase treatment was detected by SNA staining decrease.

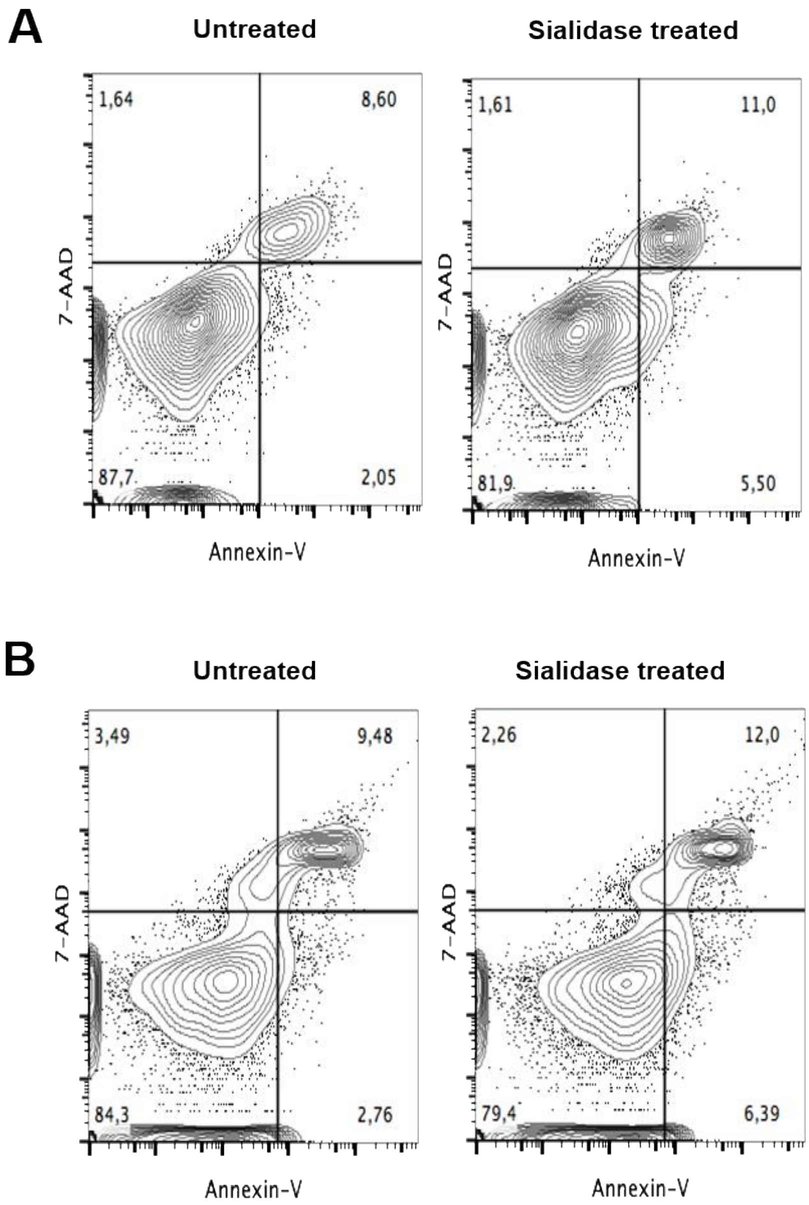

Figure S3. Assessment of cell viability after sialidase treatment of DCs. Human MoDCs (A) and murine sDCs (B) were treated with sialidase or left untreated and dual stained with Annexin-V and 7-AAD. Both human and murine DCs treated with sialidase exhibit no significant increase in cell death compared with untreated ones, suggesting that DCs can tolerate sialic acid removal relatively well and remain viable to exert their immunologic function. Importantly, sialidase treatment of DCs did not compromise DCs viability.
